# Incidence and predictors of major adverse drug events among drug-resistant tuberculosis patients on second-line anti-tuberculosis treatment in Amhara regional state public hospitals; Ethiopia: a retrospective cohort study

**DOI:** 10.1186/s12879-019-3919-1

**Published:** 2019-03-27

**Authors:** Mehari Woldemariam Merid, Lemma Derseh Gezie, Getahun Molla Kassa, Atalay Goshu Muluneh, Temesgen Yihunie Akalu, Melaku Kindie Yenit

**Affiliations:** Department of Epidemiology and Biostatistics, Institute of Public Health, College of Medicine and Health sciences, University of Gondar, Gondar, Ethiopia

**Keywords:** Adverse drug event, Drug resistant tuberculosis, Second-line anti-tuberculosis drugs

## Abstract

**Background:**

Second line anti-tuberculosis drugs are substantially complex, long term, more costly, and more toxic than first line anti-tuberculosis drugs. In Ethiopia, evidence on the incidence and predictors of adverse drug events has been limited. Thus, this study aimed at assessing incidence and predictors of major adverse drug events among drug resistant tuberculosis patients on second line tuberculosis treatment in Amhara Regional State public hospitals, Ethiopia.

**Methods:**

A multi-center retrospective cohort study was conducted on 570 drug resistant tuberculosis Patients. Data were entered in to EPI-Data version 4.2.0.0 and exported to Stata version 14 for analysis. Proportional hazard assumption was checked. The univariate Weibull regression gamma frailty model was fitted. Cox-Snell residual was used to test goodness of fit and Akaike Information Criteria (AIC) for model selection. Hazard ratio with 95% CI was computed and variables with *P*-value < 0.05 in the multivariable analysis were taken as significant predictors for adverse drug event.

**Results:**

A total of 570 patients were followed for 5045.09 person-month (PM) observation with a median follow-uptime of 8.23 months (Inter Quartile Range (IQR) =2.66–23.33). The overall incidence rate of major adverse drug events was 5.79 per 100 PM (95% CI: 5.16, 6.49). Incidence rate at the end of 2nd, 4th, and 6th months was 13.73, 9.25, 5.97 events per 100 PM observations, respectively. Age at 25–49 (Adjusted Hazard Ratio (AHR) = 3.36, 95% CI: 1.36, 8.28), and above 50 years (AHR = 5.60, 95% CI: 1.65, 19.05), co-morbid conditions (AHR = 2.74 CI: 1.12, 6.68), and anemia (AHR = 3.25 CI: 1.40, 7.53) were significant predictors of major adverse drug events.

**Conclusion:**

The incidence rate of major adverse drug events in the early 6 months of treatment was higher than that of the subsequent months. Age above 25 years, base line anemia, and co-morbid conditions were independent predictors of adverse drug events. Thus, addressing significant predictors and strengthening continuous follow-ups are highly recommended in the study setting.

## Background

Globally, Tuberculosis (TB) continues to be a major public health concern [1–3]. Currently, the emergence of drug resistant strains of TB is considered as a global threat to the control of TB. According to the World Health Organization (WHO) 2017 report, about 600,000 people were diagnosed with drug resistant tuberculosis (DR-TB). Globally in 2016, an estimated 4.1% of new cases and 19% of previously treated cases had DR-TB, respectively [4]. In Africa,about 2.7 and 14% of new and previously treated cases were diagnosed with DR-TB, respectively [5]. Ethiopia is one of the 30 high DR-TB burden countries with increasing concerns about the rising prevalence of DR-TB. According to a sentinel drug resistant survey conducted in 2014, Multi-drug resistant (MDR-TB) prevalence was 2.3 and 17.8% among new and previously treated cases, respectively [6].

The treatment of TB is an effective intervention in the control of TB epidemics globally. It saved 53 million lives globally between 2000 and 2016 [4]. The standard regimen for the treatment of DR-TB consists of groups of drugs, including fluoroqunolones [(Moxifloxacine (Mfx), Levofloxacin (Lfx)], injectable [(Kanamycin (Km), Amikacin (Am), and Capreomycin (Cm)], other core second-line agents [(Ethionamide (Eto), Prothionamide (Pto), Cycloserine (Cs), Linezolid (Lzd), and clofazimine (Cfz)], and some add-on agents [(Pyrazinamide (Z), Beda quinine (Bql), and Delamanid (Dlm)] [2, 7]. However, these drugs are known to cause excessive Adverse Drug Events (ADE) than first line anti-TB drugs, which in turn make the successful treatment of TB a challenge. An adverse drug event was defined as any untoward medical occurrence in a patient administered a pharmaceutical product that does not necessarily have a causal relationship to their treatment [2, 7]. Major ADEs reported in different studies on Second Line anti-TB Drugs (SLDs) include; Drug Induced Hepatitis (DIH), Electrolyte Disturbance (ED), Acute Psychosis (PS), Acute Kidney Injury (AKI), Peripheral Neuropathy (PN), and Hypothyroidism (HT) [2, 8].

Globally from 2015 cohort, only 55% of DR-TB cases were successfully treated due to the withdrawal of the drugs and poor treatment adherence associated with major adverse drug events [9]. The treatment success rate of DR-TB treatment in Ethiopia was higher than 75% [10, 11]. The problem of adverse drug events is more common in developing countries including Ethiopia [12]. Although long term and complex regimen of second-line treatment were associated with adverse drug events, there are also other possible determinants. Reports claimed that advanced age [13, 14], obesity [15], anemia [16], unhealthy behaviors [17, 18], and co-morbid conditions [13, 19] were predictors of the occurrence of adverse drug events. Furthermore, HIV co-infection was significantly associated with an increased risk for adverse drug event [20–23]. According to different clinical trials and observational studies, ADEs occurred during the treatment of first line anti-TB drugs [24–26]. However, to the best of our search, there is no study conducted on major ADEs related to second-line anti-TB drugs in Ethiopia as well as in the region. Therefore, our study aimed to assess the incidence rate of major adverse drug event and its predictors among drug resistant tuberculosis patients on second line tuberculosis treatment in Amhara Regional State public hospitals, Ethiopia.

## Methods

### Study design and setting

A multi-center retrospective cohort study was conducted from September 2010 to December 2017 among DR-TB patients in four of the nine hospitals in the region, namely University of Gondar comprehensive specialized hospital, Debremarkos referral hospital, Borumeda primary hospital, and Woldia primary hospital. A total of 715 DR-TB patients were enrolled since the start of the DR-TB treatment in the region of which 640 (90%) of the DR-TB patients received treatments in these four Treatment Initiative Centers (TICs). The remaining 75 (10%) of the DR-TB patients were treated in the other five TICs. Moreover, follow up profiles of patients served in these five TICs are usually done at nearby hospitals of the four main TICs on referral basis.

The first anti-TB treatment center was the University of Gondar comprehensive specialized hospital, which is in North Gondar administrative zone, located 750 km (km) northwest of Addis Ababa (the capital of Ethiopia). According to the 2018 population projection, the population size of Gondar town was estimated to be 351,350 (178,447 male and 172,903 female). The hospital started delivering such services since 2010 and has 28 treatment follow up centers and has served more than 351 DR-TB patients so far. The second TIC was Borumeda primary hospital located 10 km far from Dessie, the capital of South Wello and 441 km from Addis Ababa. It was established in 2013 and has 29 Treatments Follow-up Centers (TFCs) and has been serving more than 174 DR-TB patients so far.

The third TIC was Debremarkos primary hospital found in Debremarkos, the capital of East Gojjam administrative Zone. It is located 300 km from Addis Ababa, and 265 km from Bahir- Dar, (the capital of Amhara region) and is serving about 62 DR-TB patients. The fourth TIC was Woldia primary hospital which is located 506 km from Addis Ababa and 360 km from Bahir Dar. It has been serving more than 54 DR-TB patients since its establishment and has 16 TFCs.

### Population and sample

In this study, all DR-TB patients on second line anti-TB drugs enrolled in the DR-TB treatment centers of Amhara Regional State were the source population, whereas patients who enrolled under the four selected DR-TB treatment centers were the study population. All DR-TB patients on second line anti-TB drugs and had follow-ups at the University of Gondar comprehensive specialized hospital, Debremarkos referral hospital, Borumeda primary hospital, and Woldia primary hospitals were included.

Patients who had incomplete data for major adverse drug events and missed follow up profiles of serum creatinine, potassium liver transaminases and thyroid stimulating hormone tests were excluded from the study. In addition, patients with chronic kidney disease and hepatitis at baseline were also excluded.

The minimum sample required was determined using Stata 14.1 software with Cox proportional assumptions. Based on this, sample size was calculated for two predictor variables, namely HIV co-infection (HR = 5.81) and extra-pulmonary TB (HR = 2.74) from a prospective study done in Rwanda [16] (Table 1).

**Table 1:** Sample size determination

Assumptions	Probability of event	Variables	Hazard ratio	Sample size
Power = 80% Significant level (α) = 0.05 Allocation ratio 1:1 Two tailed	0.26	HIV co-infection	5.81	163
	0.26	Extra-pulmonary TB	2.74	495

Accordingly, the minimum sample size was 495; however, 640 DR-TB patients in the four TICS were considered. Finally, 570DR-TB patients enrolled on treatment initiating centers and fulfilled the inclusion criteria were included in the analysis.

### Variables of the study

The dependent variable was major adverse drug event, whereas socio-demographic variables (age, sex, marital status, residence, religion, educational status, and occupation), behavioral factors (cigarette smoking, alcohol drinking, and drug abuse), and clinical factors (TB type, baseline functional status, TB complication at baseline, history of treatment for first line anti–TB drugs, presence of TB complication, baseline Body Mass Index (BMI), base line hemoglobin level, second line anti-TB regimen, HIV co-infection, presence of other co-morbidities including diabetes mellitus, hypertension, chronic obstructive pulmonary diseases and asthma) were the independent variables.

Survival time was defined as time in months from the start of treatment to the development of any of the major adverse drug events. Event was defined as a patient who developed any of the major adverse drug events for the first time during the follow up time. Censored was defined as a DR-TB patient who didn’t develop any major adverse drug event at the end of the study, transferred out, cured, completed, failed or lost to follow up before developing any major adverse drug event. Major adverse drug events are serious adverse events that are often complicated to monitor and can be life threatening to warrant extra attention in monitoring for early detection and prompt management pertaining to the following medical conditions [4, 5, 22].

Acute kidney injury (AKI) was defined as creatinine level increased by (1.5–2.0) mg/dl above the baseline [27]. Hypokalemia was defined as at least one serum potassium value less than 3.0 mEq/L. [28] Hepatotoxicity was defined as at least five times elevated value of serum transaminase in the absence of symptoms or 3 times elevation in serum transaminases in the presence of symptoms to the upper limit of normal values [8]. Hypothyroidism was defined as at least one TSH value > 10 mIU/L after at least 3 months of second line TB treatment [8, 29]. Peripheral neuropathy was defined as a DR-TB patient with numbness, tingling or burning in the trunk or extremities; diminished or absent reflexes; nerve conduction studies consistent with peripheral neuropathy; vestibular side effects; the presence of at least 1 month of persistent, myasthenia-like syndrome, nystagmus, dizziness and/or loss of balance [4]. Psychosiswas also defined as presence of depression, anxiety, suicide, nightmares, or convulsions [5]. Anemia was defined as hemoglobin value of less than 12 mg/dl for males and less than 11 mg/dl for females. Body mass index (BMI): was defined as low when BMI < 18.5 kg/m^2^, and normal if BMI 18.5–24.99 kg/m^2^. Cigarette smoking: was recorded by asking respondents whether they have ever smoke cigarette in life history. It was dichotomized by 1 (Yes i.e., smoke cigarettes) and 0 (No smoke cigarettes). Alcohol consumption: was recorded by asking a respondents whether a respondents have ever drink alcohol or not. It was dichotomized by 1 (Yes i.e., drink alcohol) and 0 (No drink alcohol).

### Data collection procedures and quality control

The data extraction check-list was prepared in English. Eight BSc degree graduate nurses and four health officers collected the data under the supervision of the principal investigator. Two days training was given for the data collectors on the objective of the study and how to review the documents as per the data extraction check-list. Patient follow-up charts for baseline and follow-up records were identified by their DR-TB registration or card numbers. Data collectors reviewed and extracted the data from patient charts, medical notes, follow up green cards, and registries. The principal investigator supervised the data collectors closely. Data were checked for any inconsistencies, coding errors, out of range values, completeness, accuracy, clarity, missing values, and appropriate corrections were made by the principal investigator consistently on daily basis.

### Data processing and analysis

Data was entered in to EPI-Data version 4.2.0.0 and exported to stata14 software for analysis. Descriptive statistics were carried out and presented using tables, graphs, and texts. Percentages and medians were used for categorical and continuous variables, respectively. Person-time (incidence density) rate (IR) was calculated for the occurrence of adverse drug events. The Kaplan Meier (KM) curve and log rank test were used to describe survival experience among different categories. Proportional hazard assumption was checked both graphically and using the schoenfield residual test.

The Akaike Information Criterion (AIC) and the Bayesian Information Criterion (BIC) were estimated for model comparison. The hazard ratio was used as a measure of association. Parametric survival models were fitted by considering baseline hazard with different distributional assumptions. The frailty model takes into account the random effect model for time-to-event data by adding the frailty term, “Z”. Thus both uni-variable and shared frailty model was tested by considering different parametric distribution and the frailty distribution (gamma and inverse Gaussian). A more parsimonious hazard model was selected using the log likelihood ratio (LL) test and AIC. The model with the smallest AIC was considered the best fitted model. The goodness of fit was also checked by the Cox Snell residual test.

## Results

### Baseline socio-demographic characteristics of DR-TB patients

**Fig. 1:**
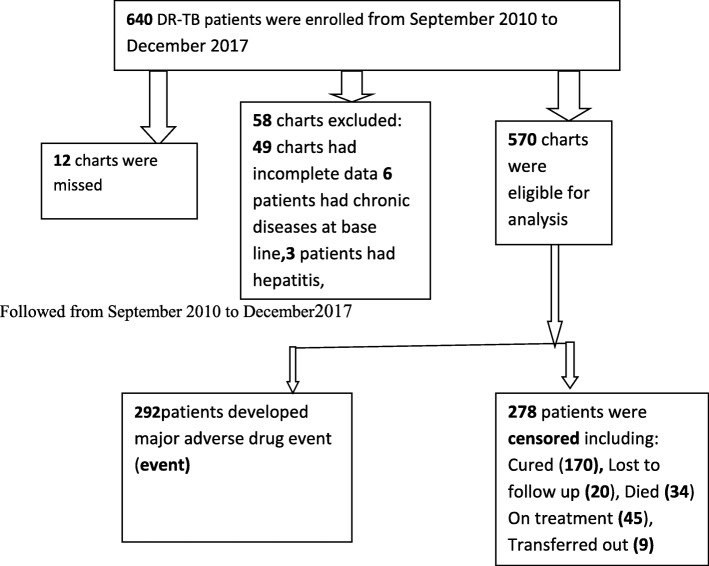
Flow chart showing a selection of DR-TB patients on second line anti-TB drugs in Amhara Regional State Public Hospitals from September 2010 to December 2017

Six hundred-forty (640) DR-TB patients were enrolled between September 2010 and December 2017 in four TIC hospitals of the region. We couldn’t access medical follow up charts of 12 patients. A total of 570 patients were included in the analysis (Fig. 1).

**Fig. 2:**
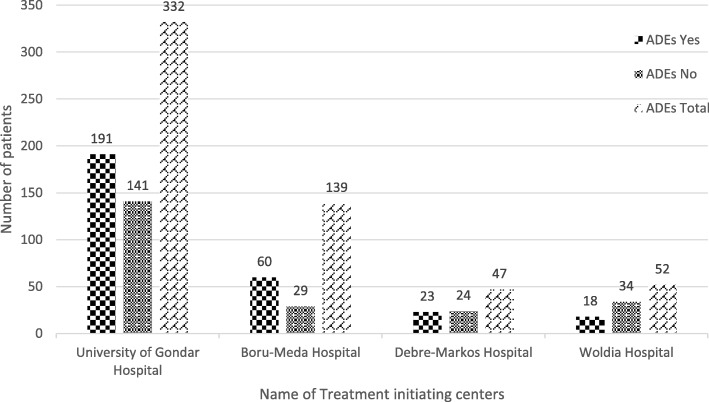
Number of drug resistant tuberculosis patients on second line anti-TB drugs stratified by TICs and major adverse drug events (ADEs) in Amhara Regional State Public hospitals, September 2010–December 2017

Majority, 332 (58.25%) of DR-TB patients were from University of Gondar Comprehensive Specialized hospital and 139 (24.39%), 47 (8.25%), and 52 (9.12%) were from D/Markos referral hospital, Borumeda primary hospital, and Wodia primary hospital respectively (Fig. 2).

The median age of patients was 28 (IQR = 23–38) years. More than half, 324 (56.84%) of the patients were male and 300 (52.63%) were urban dwellers. Nearly 50 %, 274 (48.07%) were married. The majority of the patients, 230(43.7%) were farmers followed by 169 (31.30%) unemployed. Regarding religion 472 (81.82%) was Orthodox Christian. Of the total patients, 249 (44.15%) were uneducated (Table 2).

**Table 2:** Baseline socio-demographic characteristics of DR-TB patients on second anti-TB drugs in Amhara Regional State public hospitals, Ethiopia, September 2010 to December 2017, (*N* = 570)

Variables	Frequency	Percent (%)
Age in year		
1–24	183	32.11
25–49	294	51.58
> =50	93	16.32
Sex		
Male	324	56.84
Female	246	43.16
Residence		
Urban	300	52.63
Rural	270	47.37
Educational status		
Not educated	249	44.15
Primary	154	27.30
Secondary and above	161	28.51
Occupation		
Farmer	230	42.60
Employed	141	26.11
Unemployed	169	31.30
Marital status		
Married	274	48.07
Single	180	31.58
Divorced	46	8.07
Widowed	18	3.17
Separated	30	5.26

### Baseline clinical and behavioral characteristics

Majority 502 (88%) of patients were treated for pulmonary tuberculosis. More than two thirds (70.18%) of the patients were on Z-Cm-Lfx-Pto-Cs regimen. The majority, 479 (84.04%), were treated for first line anti-TB drugs. More than a quarter (27.02%) of them were HIV co-infected, of whom 148 (96.73%) were on anti-retroviral therapy. Nearly three fourths, 402 (74.86%), had a base-line BMI less than 18.5 kg/m^2^. Seventy-seven (13.51%) had cigarette smoking and 86 (15.19%) had alcohol drinking history at base-line (Table 3).

**Table 3:** Base-line clinical and behavioral characteristics of DR-TB patients on second anti-TB drugs in Amhara Regional State public hospitals, Ethiopia, September 2010 to December 2017, (*N* = 570)

Variables	Frequency	Percent (%)
Functional status		
Working	199	35.35
Ambulatory	172	48.31
Bedridden	92	16.34
Second line regimen		
E-Z-Cm-Lfx-Eto-Cs^a^	105	18.42
Z-Cm-Lfx-Eto-Cs	65	11.40
Z-Cm-Lfx-Pto-Cs	400	70.18
HIV co-infection		
Negative	416	72.98
Positive	154	27.02
Baseline hemoglobin		
Anemic	212	37.19
Not anemic	358	62.81
Baseline body mass index(kg/m^2^)		
< 18.5	402	74.86
18.5–24.9	135	24.14
Comorbid conditions		
Yes	84	14.74
No	486	85.26
Type of TB		
Pulmonary	502	88.07
Extra-pulmonary	44	7.72
Disseminated	24	4.21
History of smoking		
Yes	77	13.51
No	493	86.49
History of alcohol drinking		
Yes	86	15.19
No	480	84.81
Drug abuse		
Yes	27	4.76
No	540	95.24

^a^*E* Ethambutol, *Lfx* Levofloxacin, *Cm* Capreomycin, *Eto* Ethionamide, *Pto* Protionamide, *Cs* Cycloserine, *Z* Pyrazinamide

### Incidence of major adverse drug events

The overall incidence rate of any major adverse drug event was 5.79 per 100 Person-Month (PM) (95% CI: 5.16, 6.49) observations. The incidence rate at the end of 2nd, 4th, and 6th months was 13.73, 9.25, and 5.97 per 100 PM observations, respectively. The sum of the whole follow-up period for all 570 DR-TB patients was 5045.09 (PM) of observation. The minimum and maximum follow-up period was 0.27 months (8 days) and 23.33 months after the initiation of second line anti-TB drugs, respectively. The median follow-up period was 8.23 months of observation (IQR = 2.77–13.69).

A total of 292 (51%) DR-TB patients initially free from any adverse drug event developed at least one major adverse drug event during the entire treatment course (95% CI: 47.11, 55.33). From all patients (292) who had major ADEs, the most common adverse drug event was hypokalemia which is 54.03% followed by renal toxicity (15.86%), acute psychosis (15.32%), peripheral neuropathy (9.14%), hepatitis (2.96%), and hypothyroidism (2.69%).

The incidence rate per 100 PM of observation in patients with and without comorbidity was 12.23 (95% CI: 9.46, 15.82) and 5.12 (95% CI: 4.50, 5.82) respectively.

Our study further estimated the incidence rate of each major adverse drug events. Hypokalemia was the most common adverse drug event with 6.72 events (95% CI: 5.82, 7.75), followed by renal toxicity 1.49 events (95% CI: 0.74, 1.67) per 100 PM observations.

**Fig. 3:**
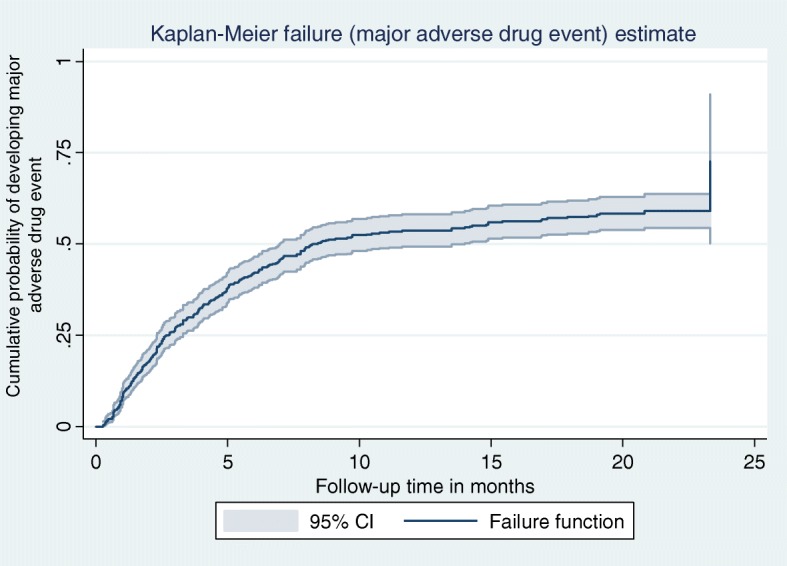
Kaplan Meier hazard function of DR-TB patients with major adverse drug event on second line anti-TB drugs in Amhara Regional State Public Hospitals from September 2010 –Dec ember 2017

The Kaplan Meier (KM) failure function was used to describe the cumulative incidence rate of adverse drug events over the follow up period (Fig. 3). The cumulative probability of survival of DR-TB patients at the end of 1st, 2nd, 3rd, and 4th months was 0.93, 0.82, 0.74, and 0.68, respectively. The median survival time was 8 months.

### Predictors of major adverse drug events

**Fig. 4:**
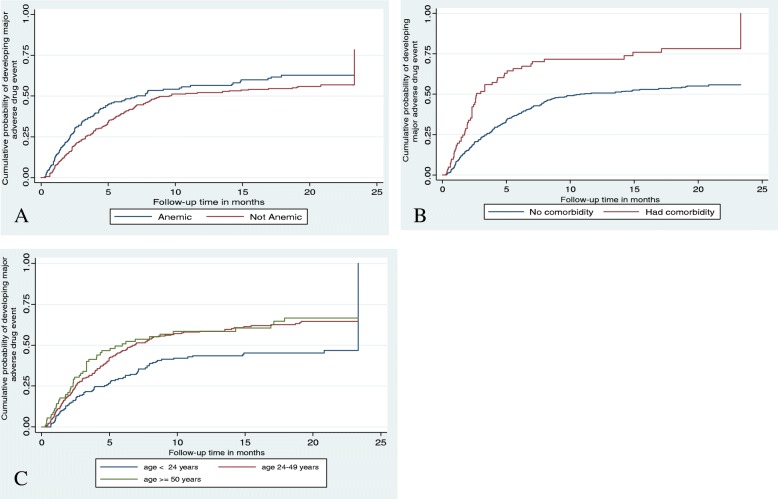
Kaplan Meier hazard curve and log-rank test by haemoglobin level (**a**), co-morbidity (**b**), and age (**c**) of DR-TB patients with major adverse drug event on second line anti-TB drugs in Amhara regional State Public Hospitals from September 2010 to December 2017

Differences in all key variables at base-line among strata were determined using the Kaplan Meier failure function and the log-rank (χ2) test. The Kaplan Meier failure function was constructed for different categorical variables. In general, the pattern of the survivorship function lying above another indicated that the group defined by the upper curve had a better survival than the group defined by the lower curve. In terms of survival experience for the occurrence of adverse drug events, there were significant variations among patients with or without co-morbidities (*p* < 0.01), and in those who were anemic and not anemic at base-line (*p* = 0.02) (Fig. 4).

The survival curve plotted in Fig. 4 indicated that the estimated hazard curves of the hospital and the log rank test used for checking the differences in hazard curve displayed. There was no overall difference between the hazard curves of the hospitals as the log rank test supported the null hypothesis (log rank Chi-square (2) = 2.3, *p* = 0.125).

### Assessing the proportional hazard assumption

The proportional hazard assumption states that the risk of failure of the participants must be the same no matter how long they are followed. The assumption checked graphically was non-overlapping. In addition, a global test based on Schoenfield residuals found that all of the covariates and the full model satisfied the proportional hazard assumption (Chi square = 10.32, *p*-value = 0.5875).

### Model comparison

After the proportional hazard assumption was checked, both semi-parametric and parametric models were fitted to estimate the survival time for developing major adverse drug events and identify the predictors among DR-TB patients on second line anti-TB drugs. The most parsimonious model was selected using AIC, BIC and LL estimation.

According to the Akaike Information Criterion, the univariate frailty with Weibull distribution and gamma frailty **(**AIC = 1453.07) model had the lowest AIC (Table 5). However, the shared frailty by treatment initiating centers had no statistically significant variance. Hence, including the frailty term indicated the absence of heterogeneity or unobserved variability among hospitals. But there was statistically significant heterogeneity among individuals **(**variance (Ɵ) = 6.45, *p* < 0.01) (Table 4).

**Table 4:** Summary of Model comparison among Cox proportional hazard model, parametric Cox- Regression models and frailty models using AIC, BIC LR criteria

Model	Baseline hazard	Frailty	Variance	AIC	BIC	Logliklihood
Cox regression	unspecified			3339.37	3382.67	--1659.68
Weibull regression	Weibull			1539.27	1591.23	− 757.63
Univariate frailty	Weibull	Gamma	6.45 (p < 0.01)	1453.07	1509.36	− 713.54
Univariate frailty	Weibull	Inverse-Gaussian	5.02(p < 0.01)	1504.51	1560.80	− 739.26
Shared frailty (by hospital)	Weibull	Gamma	0.006(*p* = 3.08	1541.02	1597.30	−757.51
Exponential regression	exponential			1566.57	1614.20	− 772.28
Gompertez regression	Gompertez			1474.27	1526.23	− 725.14

### Model diagnosis

The finding of the bivariable analysis showed that age, sex, residence, base-line hemoglobin, base-line functional status, co-morbid conditions, educational status, TB complication, and HIV co-infection were significantly associated with the occurrence of major adverse drug events. However, in the multi-variable analysis; age, co-morbid conditions, and base-line hemoglobin were statistically significant predictors of major adverse drug events (Table 5).

**Table 5:** Bi-variable and multi-variable Weibull regression gamma frailty analysis for predictors of major adverse drug events of DR-TB patients on second line anti-TB drugs in Amhara Regional State public hospitals, September 2010 to December 2017

Variables	Event	Censored	CHR (95% CI)	AHR (95% CI)
Age in year				
1–24	76	107	1	1
25–49	164	130	2.46 (1.13,5.33)	3.36 (1.36,8.28)^*^
> =50	52	41	4.45 (1.47,13.47)	5.60 (1.65,19.05)^*^
Comorbid conditions				
No	234	252	1	1
Yes^a^	58	26	2.19 (1.64,1.92)	2.74 (1.12,6.68)^*^
Sex				
Male	175	149	1	1
Female	117	129	0.68 (0.34,1.35)	1.02 (0.50,2.06)
Functional status				
Working	83	11	1	1
Ambulatory	159	113	2.09 (0.98,4.44)	1.98 (0.91,4.34)
Bed ridden	46	46	3.80 (1.43,10.07)	2.30 (0.78,6.65)
TB complication				
No	234	235	1	1
Yes	58	43	1.58 (1.18,2.11)	1.17 (0.46,2.94)
Educational status				
Above secondary	82	79	1	1
Primary	86	68	1.24 (0.92,1.68)	1.39 (0.54,3.60)
Not educated	120	129	0.97 (0.74,1.30)	0.50 (0.19,1.30)
Hemoglobin level				
Not anemic	179	179	1	1
Anemic	113	99	1.30 (1.03,1.64)	3.25 (1.40,7.53)^*^
HIV co-infection				
No	86	69	1	1
Yes	206	209	1.27 (0.98,1.63)	0.97 (0.45,2.10)

^*^Significant factors at *p*-value< 0.05

^a^Diabetes mellitus, hypertension, chronic obstructive pulmonary diseases, asthma

**Fig. 5:**
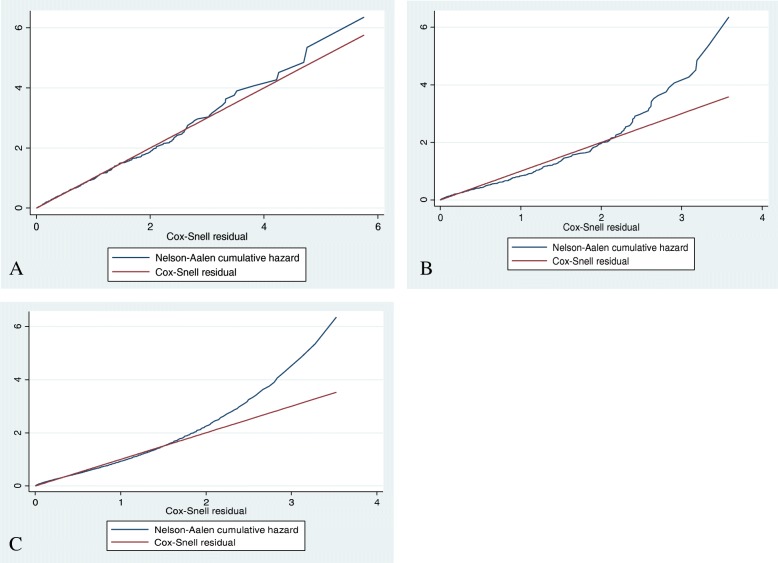
Plot of Nelsen-Allen cumulative hazard function against Cox-Snell residual obtained by fitting Weibul (**a**), Exponential (**b**) and Gompertz (**c**) models for adverse drug events of DR-TB patients on second line anti-TB drugs in Amhara Regional State Public hospitals

The Cox- Snell residuals versus the Nelson-Aalen cumulative hazard function were obtained by fitting the exponential, Weibull, and Gompertez models. It can be seen that the plot of the Nelson Aalen cumulative hazard function against the Cox-Snell residuals has a linear pattern making a straight line through the origin of the Weibull model when compared to Gompertez and Exponential models. This suggests that the Weibull regression model provided the best fit for our data set (Fig. 5).

## Discussion

This study mainly investigated the incidence and predictors of major adverse drug events among drug resistant tuberculosis patients on second line anti TB drugs in four DR-TB TICs of the Amhara Regional State public hospitals.

The overall incidence rate of major adverse drug events was 5.79 per 100 PM observations (95% CI: 5.16, 6.49). This result was higher than that of the study done in Canada [30] with 0.6 events per 100 person month and a multi-centered study conducted in five countries including Peru, Latvia, Estonia, Russia, and the Philippines [31] of 3.2 cases per 100 people month after a median follow-up time of 4 years. This could be due to the difference in health care workers ability to detect adverse drug events, variations in training on adverse events, and timely administration of ancillary drugs to prevent adverse events. In addition, the provision of appropriate dosage or regimen modification in anticipating adverse events and the use of different regimens and combinations of drugs in the latter studies might be the other justifications for the observed differences [30].

On the other hand, our result noted lower incidence of adverse drug events when compared with a study conducted elsewhere [32, 33]. The study done in China reported an overall incidence of 6.92 cases per 100 people month observations. The lower incidence of ADE in our finding when compared with the study in China was due to the differences in measuring adverse events. The study in China considered gastrointestinal disorders, arthralgia, skin disorders, ototoxicity, and hearing loss in the definition of major adverse drug events which may overestimate the incidence rate. Similarly, our finding was lower when compared with a study done in Nepal with the incidence of 8.65 cases per 100 person-month (PM) observations. Like the study in China, the study in Nepal included joint pain, rash, nausea, and vomiting in the definition of major adverse drug event which is very common to happen but less threatening to the patient [33].

In this study, more than half, 292 (51%), of the patients experienced at least one major adverse drug event during the entire treatment course (95% CI: 47.11, 55.33). This result was higher compared to that of a study done in Russia which reported a prevalence of ADE as 32.1% [31]. This could be explained as follows. Despite the use of the WHO model list of second line essential anti-TB drugs in both studies, the study in the Russia was coordinated and continuously monitored under the Green Light Committee program in that they used aggressive management strategies including altering dosages when appropriate, administration of ancillary drugs to treat adverse events, and discontinuation of some drugs. Similarly, the prevalence of major adverse drug events in this study was higher compared with those of studies in South Africa [19], Nigeria [34], with the prevalence of 34.5, and 44%, respectively. This could be due to differences in study participants and the duration of follow-up; in South Africa, the study included all admitted XDR-TB patients, while the study in Nigeria was among patients during the intensive phase of TB treatment [35].

In this study, the incidence of hypokalemia (one of the major adverse drug events) was 6.72 (95% CI: 5.82, 7.75) cases per 100 person-month. This finding was in agreement with that of a study done in Peru, reported 6.83 cases per 100 person-month [36]. The incidence rate of renal toxicity, which was 1.49 (95% CI: 0.74,1.67) cases per 100 person-month observation in this work was higher than that of a study done in Kenya [37] which showed an incidence rate of 0.43 cases per 100 person-month observations. This could be due to the study in Kenya includes only those patients ambulatory model of care while in our study we included both hospitalized and ambulatory model of care. On the other hand, the incidence rate of hypothyroidism in our study, 0.12 (95% CI: 0.09, 0.25) cases per 100 person month was lower than that of a study done in India and reported an incidence rate of 1.17 cases per 100 person months [38]. This could be justified in that 67% of patients in Indian study were co-administered both Ethionamide and Paraminosalicyclic acid but only 18.4% of cohortsin our study were taking these two drugs and it is known that these drugs are most commonly associated with increasing the risk of developing hypothyroidism.

According to the univariable Weibull regression Gamma frailty model, age (25–49, above 50 years), concurrent co-morbidity, and anemia were significant predictors of adverse drug events. Accordingly, the risk of developing adverse drug events among advanced age (25–49; and > =50 years) was higher compared to age below 24 years. This result was in line with those of studies conducted in Colombia [30], Iran [39] and Peru [13] and explained in that older patients were exposed to several drugs and could have too exhausted organs to metabolize and clear toxic substances from the body and decreased tolerance for medications and over the counter drugs, putting patients at increased risk for developing adverse drug events [7, 33].

In this study, the risk of experiencing adverse drug events in patients with co-morbidities was nearly three times higher than that of patients without any co-morbidity (AHR = 2.74, 95% CI: (1.12, 6.68). This result was supported by studies done in South Africa [19] and Peru [20], and showed patients with any type of co-morbid conditions were at increased risks for developing adverse drug events. This could be due to the fact that the pill burden and drug-drug interactions put patients at increased risk for developing different adverse drug reactions and patients with additional medical condition might have compromised immunity and poor tolerance to drugs [3, 40].

Our study noted that patients who had anemia at base-line were over three times more likely to develop adverse drug events than patients who had no anemia (AHR = 3.25 CI: 1.40, 7.53), and this was supported by a study done in Lima, Peru [13]. This can be seen as anemia as part of the clinical manifestation of tuberculosis as a result of chronic diseases predisposed patients to delayed gastro-intestinal absorption of nutrients resulting in poor tolerance to drugs and development of adverse drug events [41]. Moreover, occult gastro-intestinal bleeding caused by tuberculosis by itself, leads to anemia and poor nutritional status and increased risk for developing adverse drug events [42].

Although attempts were made to indicate major adverse drug events among drug-resistant tuberculosis patients, some limitation were not ruled out. The retrospective nature of the study made follow up data incomplete like serum profiles. Moreover, we couldn’t find the treatment profile of patients with other co-morbid conditions and drugs which may affect the occurrence of major adverse events.

## Conclusion

The incidence of adverse drug events among MDR-TB patients was decreasing through the subsequent months of follow up. Age above 25 years, base-line anemia, and co-morbid medical conditions were independent predictors associated with increased risks of adverse drug events in patients on second line anti-TB drugs. Patients with these risk factors need to be cautiously monitored during the anti-TB therapy. Moreover, since adverse drug events associated with second line anti-TB drugs are an important determinants of treatment outcomes, estimating the incidence and associated factors of adverse drug events provides crucial information for clinicians to identify high risk groups and adjust their drug regimen accordingly for better tolerance to anti-TB drugs to improve the quality of life of patients and to make treatment outcome successful.

## Abbreviation

ADE: Adverse drug event
ADR: Adverse drug reaction
AHR: Adjusted hazard ratio
AIDS: Acquired immune deficiency syndrome
AKI: Acute kidney injury
BMI: Body mass index
CI: Confidence interval
DOTs: Directly observed therapies
DR-TB: Drug resistant tuberculosis
ED: Electrolyte disturbance
HA: Hazard ratio
HIV: Human immune deficiency virus
INH: Isoniazid
KM: Kaplan meier
MADE: Major adverse drug event
MTB: Mycobacterium tuberculosis
RIF: Rifampicin
SDG: Sustainable development
SLD: Second line drugs
TB: Tuberculosis
TICs: Treatment Initiating Centers
WHO: World Health Organization
